# Validation of the German version of the SarQoL^®^ questionnaire in sarcopenic and probable sarcopenic patients

**DOI:** 10.1007/s40520-024-02870-z

**Published:** 2024-11-11

**Authors:** Sebastian Martini, Christopher Held, Sabine Schluessel, Olivia Tausendfreund, Anna Schaupp, Michaela Rippl, Benedikt Schoser, Ralf Schmidmaier, Michael Drey

**Affiliations:** 1 Department of Medicine IV, LMU University Hospital, LMU Munich, Munich, Germany; 2 Department of Neurology and Friedrich-Baur-Institute, LMU University Hospital, LMU Munich, Munich, Germany

**Keywords:** Sarcopenia, Health-related quality of life, SarQoL^®^, Validation, Older adults

## Abstract

**Background:**

The German version of the SarQoL^®^, a sarcopenia-specific quality of life (QoL) questionnaire, has not been validated hindering its widespread use. This study aimed to evaluate the psychometric properties of the German SarQoL^®^.

**Methods:**

Via a cross-sectional study participants were recruited in two geriatric outpatient facilities and one acute geriatric ward in Munich (Germany). Sarcopenia and probable sarcopenia were diagnosed with the European Working Group on Sarcopenia in Older People (EWGSOP2) algorithm. From 185 participants (age 79.8 ± 6.1), 77 had probable sarcopenia, and 51 had sarcopenia. Participants completed the SarQoL^®^ and the European Quality-of-Life 5-Dimension (EQ-5D) questionnaires. The validation included examination of the discriminative power, construct validity, internal consistency, test-retest reliability, and floor/ceiling effects.

**Results:**

Lower SarQoL^®^scores for sarcopenic (p = 0.002) and probable sarcopenic subjects (p < 0.001) compared to controls indicated good discriminative power. Consistent construct validity was found for sarcopenic subjects: moderate to high correlations with domains capturing similar constructs of the EQ-5D: ‘Activities of daily living’ (r = -0.58, p < 0.001), ‘Mobility’ (*r* = -0.72, *p* < 0.001) and low correlations with domains related to different constructs like ‘Pain’ (*r* = -0.32, *p* < 0.022). Similar correlations were found for probable sarcopenic subjects. The Cronbach’s alpha was 0.8. Test-retest reliability was excellent (intraclass coefficient correlation of = 0.96; 95% CI = 0.91–0.99), and no floor/ceiling effects were observed.

**Conclusion:**

QoL was similarly reduced in both patient cohorts compared to controls. The German SarQoL^®^ is a valid and reliable instrument for measuring QoL in patients > 65 years of age with sarcopenia and probable sarcopenia and can now be used in epidemiological studies and clinical trials in a German-speaking population.

**Trial registration:**

German Clinical Trials Register (DRKS)-ID: DRKS00020504 (March 12th, 2021) .

## Background

Rosenberg originally introduced the concept of sarcopenia as a geriatric syndrome associated with adverse effects on function, quality of life, and survival [1]. According to the European Working Group on Sarcopenia in Older People (EWGSOP2) the current definition of sarcopenia includes the impairment of muscle mass, strength, and muscle function [2]. EWGSOP2 added criteria for identifying patients with probable sarcopenia that would suffer from reduced grip strength or were taking > 15 s to perform five chair stand repetitions but still had an appendicular lean mass (ALM) within the normal range. Confirmed sarcopenia is defined as having ALM relative to a squared body height of < 5.5 kg/meters squared (kg/m2) for women and < 7 kg/m2 for men.

The prevalence of sarcopenia among healthy men and women aged ≥ 65 years in Europe ranged between 11.1% and 20.2%. These numbers are most likely to increase soon, particularly in higher age groups [3].

Albeit sarcopenia is already associated with a higher mortality and hospitalization rate compared to non-sarcopenic older patients, it also has detrimental effects on the functional ability and life satisfaction of patients, resulting in a lower quality of life [4]. Patients’ subjective experiences as perceived QoL, can be assessed by Patient Reported Outcome Measures (PROMs) that can help to understand the differences that interventions in sarcopenia make to people’s perceived QoL with the ultimate goal to improve it [5].

Several studies investigated QoL in sarcopenic patients. Most of these studies used generic PROMs like the 36-item Short-Form Health Survey (SF-36) and the European Quality-of-Life 5-Dimension (EQ-5D) questionnaire [6, 7]. As these questionnaires do not cover all areas of muscle dysfunction and the resulting life dissatisfaction in sarcopenic patients, they at least partially fail to uncover the reduced QoL in sarcopenic patients [8, 9]. As a consequence, Beaudart and colleagues developed and validated the sarcopenia-specific questionnaire SarQoL^®^, which displays a higher sensitivity and construct validity for changes in QoL in sarcopenia over time compared to the aforementioned generic questionnaires [8]. It consists of 55 items translated into 22 questions, covering 7 domains of health-related dysfunction: Physical and mental health, Locomotion, Body composition, Functionality, Activities of daily living, Leisure activities, and Fears. The SarQoL^®^ is the only validated questionnaire for sarcopenic patients’ QoL.

To date, researchers have translated the SarQoL^®^ into 35 languages and its properties have been validated for 18 of those 35 languages: Brazilian [10], Chinese [11], Dutch [12], English [13], French [8], Greek [14], Hungarian [15], Korean [16], Lithuanian [17], Persian [18], Polish [19], Romanian [20], Russian [21], Serbian [22], Spanish [23], Taiwanese [24], Turkish [25], and Ukrainian [26]. On their well-maintained website: https://www.sarqol.org access to interpretation tools and the available translated versions of the SarQoL^®^ is granted.

The original French version of the SarQoL^®^ was translated and cross-culturally adapted into German language using the recommended best practice protocol for translation (Dr Lena Dasenbrock, Oldenburg, Germany), but to date, no research has been published using the German SarQoL^®^ questionnaire. This is most likely due to the lack of validation of the SarQoL^®^. A thorough validation process is required beforehand to demonstrate adequate reliability and validity in a representative sample cohort [27].

The current study aims to evaluate the discriminative power, construct validity, internal consistency, test-retest reliability, and floor- and ceiling effects of the German translation of the questionnaire to finally provide a validated version of the SarQoL^®^ so that it can be used in the German-speaking population.

## Methods

### Participants

Between January 2018 and December 2022, 185 subjects were recruited from inpatient and outpatient facilities of the geriatric department of the Ludwig-Maximilians-University (LMU) hospital in Munich (Germany). The Ethics Committee of the Medical Faculty of the LMU approved the study (Project no.: 23–0376 (June 5th, 2023)). The inclusion criteria were as follows: age of 65 years and older, German proficiency, and the ability to provide informed consent. Exclusion criteria were moderate to severe dementia, known neuro-muscular diseases (myasthenia gravis, Parkinson’s disease, Amyotrophic Lateral Sclerosis, polio), immobility, and amputated limbs. All study participants gave informed consent. We collected and stored anonymized patient data in a secure open-source database (www.libreclinica.org). All subjects received a geriatric assessment, including functional tests that allowed for the characterization of the study population and the diagnosis of probable sarcopenia or sarcopenia or exclusion of these two.

### Sarcopenia measures

Sarcopenia and probable sarcopenia were diagnosed according to EWGSOP2’s definition. Handgrip strength was measured by a calibrated hydraulic handheld dynamometer (JAMAR, Los Angeles, CA) and appendicular lean mass was measured using calibrated dual-energy X-ray absorptiometry (Lunar Prodigy, GE Healthcare Technologies, USA). Skeletal muscle index (SMI) was calculated by dividing the sum of the appendicular lean mass of both the arms and legs by the square of the body height [2].

### Quality of life questionnaires

Health-related quality of life was assessed by using the two questionnaires SarQoL^®^ and EQ-5D.

#### SarQoL®

The SarQoL^®^ consists of 55 items assessed over 22 questions using a Likert scale of three, four or five points and multiple-choice questions. The questionnaire includes seven main domains of dysfunction that frequently occur in sarcopenia. These domains are ‘Physical and Mental Health’ (D1–8 items), ‘Locomotion’ (D2–9 items), ‘Body composition’ (D3–3 items), ‘Functionality’ (D4–14 items), ‘Activities of Daily Living’ (D5–15 items), ‘Leisure Activities’ (D6–2 items), and ‘Fears’ (D7–4 items). Each score of the seven domains and the total score can reach point values between 0 and 100 points. A higher score reflects a higher quality of life.

#### EuroQoL-5-dimension (EQ-5D) and EQ-5D visual analogue scale

The EQ-5D questionnaire is a not disease-specific instrument for evaluating health-related quality of life aspects. It is recommended by the developers of the SarQoL^®^ for the validation of translated versions of the SarQoL^®^, The EQ-5D consists of two parts. The first part of the EQ-5D is a descriptive system with five domains: ‘Mobility’, ‘Self-care’, ‘Usual activities’, ‘Pain/discomfort’ and ‘Anxiety/depression’. Results are then converted into the EQ-5D-Index. A higher index reflects a higher health-related quality of life. The second part of the EQ-5D consists of a visual analog scale on which patients rate their overall health on a scale from 0 to 100 (worst conceivable to best conceivable state of health) [28].

### Validation of the German version of the SarQoL®

With the introduction of the SarQoL^®^, the developers provided guidelines for conducting the translation, adaptation and validation processes in a coherent and homogenous manner, which have helped developing appropriate versions of the SarQoL^®^ questionnaire for aforementioned languages [8]. For the validation process of the SarQoL^®^ a sample size of 50 non-sarcopenic and 50 sarcopenic patients, matched for age and gender if possible, is recommended [29]. The following psychometric properties of the SarQoL^®^ in the German version were examined in this study: validity (discriminative power, construct validity), reliability (internal consistency, test-retest reliability) and floor/ceiling effects.

### Discriminative power

Discriminative power describes the ability of a questionnaire to discriminate between subjects with different stages of diseases. We compared total scores and individual scores of the seven domains of the SarQoL^®^ between non-sarcopenic and sarcopenic and between non-sarcopenic and probable sarcopenic subjects. For the two-group comparison (non-sarcopenic versus sarcopenic and non-sarcopenic versus probable sarcopenic subjects), a logistic regression analysis (adjusted for age and gender) was performed.

### Construct validity

Construct validity assesses how appropriately a test measures the concepts it was designed to evaluate. In the case of the SarQoL^®^, the hypotheses refer to high or low correlations with domains of other health-related QoL-PROMs that capture similar or different constructs, respectively [30]. For this, the EQ-5D was filled out by all study participants.

Construct validity is assessed by examining convergent and divergent validity. For this purpose, Spearman rank correlations were calculated for the sarcopenia and probable sarcopenia cohorts. To test convergent validity, we conducted correlation analyses between the total scores of SarQoL^®^ and the following domains of the EQ-5D: ‘Mobility,’ ‘Usual activities,’ EQ-5D Visual Analog Scale (VAS), EQ-5D Index, that measure similar constructs. Divergent validity was tested by calculating correlations between the SarQoL^®^ score and domains of the EQ-5D that capture different constructs (‘Self-care,’ ‘Pain/discomfort,’ ‘Anxiety/depression’).

### Internal consistency

The homogeneity of the SarQoL^®^ was measured using Cronbach’s alpha coefficient. It is calculated as a measure of the strength of the reliability of the seven domains [30]. A Cronbach’s alpha greater than 0.70 indicates a high level of internal consistency [8]. In addition, we examined the correlation of each of the seven domains with the total score using Spearman rank correlation. A correlation > 0.81 is considered excellent, between 0.61 and 0.80 is very good, between 0.41 and 0.60 is good, between 0.21 and 0.4 is acceptable, and less than 0.20 is inadequate [8].

### Test-retest reliability

For testing the reliability of the SarQoL^®^ in repeated sessions, 20 consecutive study participants were asked to answer the SarQoL^®^ questionnaire again three days after the initial test. Only study participants with no health changes within the three days before the retest were used for the analysis. We calculated the intraclass coefficient correlation (ICC) to test the reliability between the test scores and the retest. An ICC above 0.7 is considered acceptable [13].

### Floor and ceiling effects

Floor and ceiling effects are present when a high percentage of the population score the lowest or highest. They should be smaller than 15%, otherwise, they are considered significant [5].

### Statistical analysis

The study population was divided into cohorts according to their sarcopenia status (non-sarcopenic, probable, and sarcopenic). Significance testing between the three groups was performed using the chi-squared test for qualitative variables and an analysis of variance (ANOVA) for quantitative variables. A p-value of < 0.05 was considered statistically significant. Unless stated otherwise, descriptive statistics are presented as mean and standard deviation or percentages. An age- and gender-adjusted logistic regression analysis was performed for the two-group comparisons. All analyses were performed using SPSS statistical software version 29 (IBM-SPSS Inc., Chicago, II, USA).

## Results

### Study cohort

The mean age of the 185 study participants was 79.8 ± 6.1 years, and 141 (76.2%) were female. According to the EWGSOP2 consensus definition, 51 subjects were sarcopenic, 77 subjects had probable sarcopenia, and 57 subjects were non-sarcopenic. The characteristics of the study population (*n* = 185) are listed in Table 1. The 3-group comparison of sarcopenic, probable sarcopenic, and not sarcopenic patients revealed significant differences between them regarding all recorded clinical characteristics among them (age, gender, Body Mass Index (BMI), Mini Mental State Examination (MMSE), Charlson Comorbidity Index (CCI), number of drugs, Short Form-Mini Nutritional Assessment (SF-MNA), SMI, hand grip strength and gait speed) except the Beck Depression Inventory-II (BDI-II) [31]. Upon detailed inspection, 40 of the 51 subjects with sarcopenia had gait speeds below or equal to 0.8 m/s. Therefore and based on gait speed alone, these 40 subjects would thereby qualify for the attribute “severe” according to EWGSOP2’s criteria. But as other data that determine the severity of sarcopenia i.e. the Short Physical Performance Battery (SPPB) were not available, we were unable make a definitive statement on the severity status for all sarcopenic patients.

**Table 1:** Clinical characteristics of the study participants

	All (*n* = 185)	No sarcopenia (*n* = 57)	Probable sarcopenia (*n* = 77)	Sarcopenia (*n* = 51)	*p*-value
Age [Years]	79.8 ± 6.1	76.8 ± 4.8	81.1 ± 6.0	81.3 ± 6.4	**< 0.001**
Women, n (%)	141 (76.2)	50 (87.7)	64 (83.1)	27 (52.9)	< 0.001*
BMI [kg/m^2^]	25.8 ± 5.0	26.8 ± 4.6	27.6 ± 4.9	21.8 ± 3.1	**< 0.001**
MMSE [/30 points]	27.0 ± 2.6	28.4 ± 2.0	27.5 ± 2.1	25.9 ± 3.0	**< 0.001**
BDI-II [/63 points]	11.7 ± 7.8	12 ± 9.5	11.9 ± 7.1	11.1 ± 6.8	0.793
CCI [points]	1.9 ± 2.1	1.2 ± 1.5	1.7 ± 1.9	2.8 ± 2.6	**< 0.001**
Number of drugs	7.7 ± 3.4	6.5 ± 2.9	7.9 ± 3.5	8.6 ± 3.4	**0.004**
SF-MNA [/14 points]	11.2 ± 2.8	12.5 ± 1.7	11.8 ± 2.4	8.7 ± 3.0	**< 0.001**
SMI [kg/m^2^]	6.6 ± 1.2	6.9 ± 1.1	7.0 ± 1.0	5.5 ± 0.8	**< 0.001**
Hand grip strength [kg]	22 ± 7.7	26.3 ± 6.8	21 ± 8.1	18.6 ± 5.7	**< 0.001**
Gait speed [m/s]	0.82 ± 0.38	1.2 ± 0.44	0.72 ± 0.23	0.60 ± 0.23	**< 0.001**

Data presented as mean ± standard deviation or number (%). BMI, Body Mass Index; MMSE, the Mini Mental State Examination for dementia screening; BDI, Beck Depression Inventory-II; CCI, Charlson Comorbidity Index; SF-MNA, the Mini Nutritional Assessment Short Form; SMI, Skeletal muscle Index. p-Values < 0.05 are indicated in bold. p-Value: ANOVA. *p-Value: Chi-squared test.

### Psychometric properties of the German version of the SarQoL®

#### Discriminative power for sarcopenic patients

The sarcopenic patients reported a lower quality of life in the SarQoL^®^ total score (51.6 ± 12.9) compared to the non-sarcopenic patients (62.6 ± 15.5) (Table 2). Logistic regression adjusted for age and sex showed an odds ratio (OR) of 0.943 (95% CI 0.909–0.979) (Table 3). All seven domains presented lower scores of the sarcopenic subjects compared to the non-sarcopenic ones, but only achieved significance levels in domains 2, 4, and 5 (Tables 2 and 3).

**Table 2:** Results of the SarQoL^®^ questionnaire for sarcopenic, probable sarcopenic and non- sarcopenic patients. Data presented as mean ± standard deviation

	No sarcopenia (*n* = 57)	Probable sarcopenia (*n* = 77)	Sarcopenia (*n* = 51)
Total Score	62.6 ± 15.5	51.1 ± 12.6	51.6 ± 12.9
D1 Physical and Mental Health	64.0 ± 17.0	57.3 ± 13.7	59.8 ± 16.0
D2 Locomotion	60.0 ± 24.7	45.0 ± 18.5	47.7 ± 20.0
D3 Body composition	61.4 ± 17.7	57.4 ± 16.1	58.0 ± 15.2
D4 Functionality	68.7 ± 16.1	55.8 ± 14.5	54.7 ± 13.7
D5 Activities of daily living	59.8 ± 16.3	45.5 ± 15.6	47.0 ± 15.4
D6 Leisure activities	45.9 ± 23.5	44.7 ± 22.8	33.9 ± 21.1
D7 Fears	65.1 ± 22.8	62.2 ± 21.5	58.4 ± 18.4

**Table 3:** Discriminative power of the SarQoL^®^ questionnaire for sarcopenic patients * adjusted for age and sex. p-Values < 0.05 in bold. OR = odds ratio; CI = confidence interval

	Sarcopenia vs. no sarcopenia (*n* = 51)		
	OR	95% CI	*p*-Value*
Total Score	0.943	0.909–0.979	**0.002**
D1 Physical and Mental Health	0.975	0.949–1.002	0.074
D2 Locomotion	0.977	0.957–0.998	**0.031**
D3 Body composition	0.985	0.959–1.012	0.273
D4 Functionality	0.928	0.892–0.966	**< 0.001**
D5 Activities of daily living	0.947	0.916–0.980	**0.002**
D6 Leisure activities	0.980	0.960–1.001	0.062
D7 Fears	0.993	0.971–1.016	0.548

#### Discriminative power in probable sarcopenic patients

Probable sarcopenic participants also had lower SarQoL^®^ total score compared with the non-sarcopenic cohort (51.1 ± 12.6 versus 62.6 ± 15.5) (Table 2). There were also significantly lower scores in domains D1, D2, D4, and D5 (Table 2 and Supplemental Table 1). The odds ratio (OR) of the adjusted logistic regression for the total score was 0.945 (95% CI 0.918–0.973).

### Internal consistency

The Cronbach’s Alpha as a measure for internal consistency of the SarQoL^®^ was 0.80. All seven domains correlated significantly and positively, ranging from *r* = 0.21, *p* = 0.005 (domain D6 ‘Leisure activities’) to *r* = 0.90, *p* < 0.001 (domain D4 ‘Functionality’) with the SarQoL^®^ total score (Table 4).

**Table 4:** Internal consistency by results of the correlation between each domain and the total score of the SarQoL^®^

	Correlation All *n* = 185	
	*r*	*p*-Value
D1 Physical and Mental Health	0.77	**< 0.001**
D2 Locomotion	0.89	**< 0.001**
D3 Body composition	0.64	**< 0.001**
D4 Functionality	0.90	**< 0.001**
D5 Activities of daily living	0.87	**< 0.001**
D6 Leisure activities	0.21	**0.005**
D7 Fears	0.39	**< 0.001**

Spearman’s correlation. p-Values < 0.05 in bold.

### Construct validity in sarcopenic patients

Results of the construct validity of the sarcopenia cohort are presented in Table 5. The SarQoL^®^ total score showed moderate to high positive correlations with those domains of the EQ-5D that capture similar constructs, such as ‘Mobility’ (r = -0.72, p < 0.001), ‘Activities of daily living’ (*r* = -0.58, *p* < 0.001) and the EQ-5D Index (*r* = 0.62, *p* < 0.001). The EQ-5D VAS negatively correlated with the SarQoL^®^ total score (*r* = 0.37, *p* < 0.01).

For the divergent validity, low to moderate negative correlations between the SarQoL^®^ total score and domains of the EQ-5D questionnaire that capture different constructs, such as EQ-5D ‘Pain/ discomfort’ (r = -0.32, p < 0.022) and EQ-5D ‘Anxiety/ depression’ (*r* = -0.32, *p* < 0.024) were found. The EQ-5D domain ‘Self-care’ correlated highly and negatively with SarQoL^®^ total score (*r* = -0.65, *p* < 0.001).

**Table 5:** Construct validity for sarcopenia by correlation of the total SarQoL^®^ score and the individual domains of EQ-5D

Convergent validity (*n* = 51)	*r*	*p*-Value
EQ-5D mobility	-0.72	**< 0.001**
EQ-5D usual activity	-0.58	**< 0.001**
EQ-VAS	0.37	**0.01**
EQ-5D-index	0.62	**< 0.001**
Divergent validity		
EQ-5D self-care	-0.65	**< 0.001**
EQ-5D pain/ discomfort	-0.32	**0.022**
EQ-5D anxiety/ depression	-0.32	**0.024**

Spearman’s correlation. P-Values < 0.05 in bold.

### Construct validity in probable sarcopenic patients

Similar results as in the sarcopenia cohort were found in the probable sarcopenia cohort (Supplemental Table 2). The high correlations of the domains EQ-5D ‘Mobility’, EQ-5D ‘Activities of daily living’, EQ-5D VAS and EQ-5D Index (from *r* = -0.65 to 0.62, all: *p* < 0.001) with the SarQoL^®^ total score confirm convergent validity of the SarQoL^®^.

For the divergent validity, low to moderate negative correlations between the SarQoL^®^ total score and domains of the EQ-5D questionnaire that measure different constructs, such as EQ-5D ‘Self-care’ (r = -0.48, p < 0.001), EQ-5D ‘Pain/ discomfort’ (r = -0.33, p < 0.004) and EQ-5D ‘Anxiety/ depression’ (*r* = -0.22, *p* = 0.056) were found.

#### Test-retest reliability

After a 3-day interval, 20 study participants who reported no change in their health status during this period were asked to fill in the questionnaire a second time to evaluate the test-retest reliability of the SarQoL^®^ questionnaire. The total score of the SarQoL^®^ questionnaire had an ICC of 0.96 (95% CI: 0.91–0.99). The ICC for the individual domains ranged from 0.51 to 0.93 (95% CI: -0.1–0.97) (Supplemental Table 3).

### Floor and ceiling effects

Neither floor nor ceiling effects were present, as none of the 185 participants achieved the lowest (0 points) or the highest (100 points) score in the total score or in any of the individual domains of the SarQoL^®^.

## Discussion

We investigated the psychometric properties of the German version of the SarQoL^®^ questionnaire and found significantly lower SarQoL^®^ scores in sarcopenic and probable sarcopenic patients compared to non-sarcopenic controls. SarQoL^®^ scores in sarcopenic and probable sarcopenic patients were almost identical, indicative of a similarly lower QoL in both patient cohorts. While the overall SarQoL^®^ score is lower in patients vs. controls, these differences achieved statistical significance only for the individual domains D2 ‘Locomotion’, D4 ‘Functionality’ and D5 ‘Activities of daily living’ for the sarcopenic cohort, with the addition of D1 ‘Physical and mental health’ as a significantly different domain for the cohort with probable sarcopenia. Thereby, the SarQoL^®^ displayed good discriminative power for both sarcopenic and probable sarcopenic patients.

The German SarQoL^®^ questionnaire also displayed a similar level of internal consistency as previous validations for other languages [10, 12–16, 18, 19, 21–26].

### Internal consistency

The German version of the SarQoL^®^ has also shown a high internal consistency (Cronbach’s alpha of 0.8), comparable with other validation studies, where a value between 0.7 and 0.95 is interpreted as adequate internal consistency [32]. Among the individual domains, the correlation coefficients ranged from 0.21 to 0.90, with domain D6 ‘Leisure activities’ displaying the weakest correlation but still achieving statistical significance. The weaker correlation for this domain is in line with results from previous validations in other languages [33]. It might be due to the fact that domain D6 only consists of 2 questions compared to the more extensive catalog of questions for the other domains.

### Construct validity and test-retest reliability

Construct validity is also equally acceptable for both sarcopenic and probable sarcopenic patients. Here we could show that the overall QoL score of the SarQoL questionnaire significantly correlated with similar domains linked to muscle performance like mobility (− 0.65 for probable sarcopenia, -0.72 for sarcopenia) of the EQ-5D questionnaire. We can thus confirm the convergent validity of the SarQoL questionnaire.

As test–retest reliability analysis showed excellent results and neither floor nor ceiling effects were present, the German version of the SarQoL^®^ questionnaire’s validation process is complete.

### Strengths and limitations

Our study has several strengths. Firstly, we included in our validation process representative geriatric cohorts of sufficient size for each entity of EWGSOP2´s definition of sarcopenia (77 probable sarcopenic, 51 sarcopenic). Previous validations in other languages, on the other hand, sometimes did not differentiate between probable sarcopenia and sarcopenia, either because EWGSOP2 did not exist yet [13] or since one cohort was underrepresented [15]. Secondly, for the estimation of muscle mass, we applied the more accurate albeit more expensive method dual-energy X-ray absorptiometry (DXA) instead of bioimpedance analysis (BIA).

Our study has some limitations. The recruited sample was not random and it is, therefore possible that our cohort’s characteristics are different from a larger population of German-speaking sarcopenic or presarcopenic individuals. However, the overall QoL score measured by the SarQoL^®^ questionnaire is within the range found in other validation studies [20, 25]. Other methodological limitations are related to the recruitment process, such as our male-to-female ratio (76.2% female), which reflects the gender imbalance in geriatric outpatient facilities and acute geriatric wards. With a BMI of 21.8 ± 3.1 kg/m2, our sarcopenic cohort has a lower BMI than the probable sarcopenic cohort. Although this is typical finding [34], we cannot exclude that an overlapping pathophysiology like malnutrition or cachexia can at least partially be at play here [35]. With three days, the test-retest timeframe was short. This reflects the shorter periods of patients’ stays in a maximum-care university hospital compared to other healthcare settings. While other validations of the SarQoL^®^ omitted the test-retest completely [15], we decided to include this important part of the validation process despite the shorter timeframe.

While previous SarQoL^®^ validation studies often use the EQ-5D descriptive system without the visual analog scale (EQ-5D VAS) as a generic PROM to test for construct validity, we decided to include the EQ-5D VAS as it has been done previously [26], but omitted the conduction of another PROM like the SF-36 questionnaire.

## Conclusions

QoL was similarly reduced in patients > 65 years of age with sarcopenia and probable sarcopenia compared to controls. The German SarQoL^®^ is a valid and reliable instrument for measuring QoL in patients > 65 years of age with sarcopenia and probable sarcopenia and can now be used in epidemiological studies and clinical trials in a German-speaking population.

## Data Availability

No datasets were generated or analysed during the current study.

## Abbreviations

ALM: Appendicular Lean Mass
ANOVA: Analysis Of Variance
BDI-II: Beck Depression Inventory-II
BIA: Bioimpedance Analysis
BMI: Body Mass Index
CCI: Charlson Comorbidity Index
DXA: Dual-energy X-ray Absorptiometry
EQ-5D: European Quality-of-Life 5-Dimension
EQ-5D VAS: European Quality-of-Life 5-Dimension Visual Analog Scale
EWGSOP2: European Working Group on Sarcopenia in Older People 2
ICC: Intraclass Coefficient Correlation
MMSE: Mini Mental State Examination
OR: Odds Ratio
PROMs: Patient Reported Outcome Measures
QoL: Quality Of life
SarQoL®: Sarcopenia-specific Quality of Life
SF-36: 36-Item Short-Form Health Survey
SF-MNA: Mini Nutritional Assessment (Short Form)
SMI: Skeletal Muscle Index
SPPB: Short Physical Performance Battery
